# An Extensive Review on Imaging Diagnosis Methods in Takotsubo Syndrome

**DOI:** 10.31083/j.rcm2410300

**Published:** 2023-10-20

**Authors:** Catalina Paraschiv, Livia Paduraru, Serban Balanescu

**Affiliations:** ^1^University of Medicine and Pharmacy “Carol Davila”, 050474 Bucharest, Romania; ^2^Cardiology Department, Elias Emergency Univeristy Hospital, 011461 Bucharest, Romania

**Keywords:** acute coronary syndrome, echocardiography, coronary angiography, cardiac magnetic resonance

## Abstract

Takotsubo Syndrome (TS) is an acute, reversible cardiac dysfunction, with complex, not entirely understood pathophysiology and heterogeneous clinical picture. Imaging methods each have a crucial role in the diagnosis, in-hospital management, short term and long term follow up. Coronary angiography needs to be performed, especially in the setting of a suspected acute coronary syndrome, in order to rule out coronary artery disease. Echocardiography plays a central role both in the acute and the chronic phase. It is the first imaging investigation performed in patients with TS, valuable to diagnose systolic dysfunction, the wall motion pattern and early complications. Cardiac magnetic resonance tissue characterization provides an essential role in the differential diagnosis of TS with other non-ischemic causes of systolic dysfunction. This review focuses on the imaging methods and the important part they play in the complex management of the disease.

## 1. Introduction

Takotsubo syndrome (TS) is generally regarded as a transient left ventricular systolic dysfunction caused by a precipitating factor, be it physical or psychological. The name Takotsubo comes from the resemblance of the end systolic shape of the left ventricle (LV) with a Japanese octopus trap, characterized by a narrow neck and a round apex. This is recognized as the classic Takotsubo pattern — the apical variant. This clinical instance was first described in Japan in 1990 [1]. The clinical resemblance between TS and acute coronary syndromes (ACS) makes it of utmost importance for clinicians to differentiate them in order to apply the best treatment.

International Takotsubo Diagnostic Criteria (InterTAK), Mayo and European Heart Failure Association of the European Society of Cardiology diagnosis criteria include transient regional wall motion abnormalities of the left (and occasionally right) ventricle in the presence of electrocardiographic dynamic changes and positive troponin and brain natriuretic peptide (BNP) levels without a culprit coronary artery lesion [2, 3, 4, 5]. Usually, a physical or an emotional trigger preceding the event can be identified but it is not mandatory for the diagnosis. Classically known as the “broken heart syndrome”, the identifiable emotional triggers may also be of a happy nature [6, 7]. Frequently described physical triggers are sepsis and neurological disease – mainly subarachnoid hemorrhage or epileptic convulsion [8, 9, 10]. An acute, transient systolic dysfunction, called neurogenic cardiomyopathy or neurogenic distress syndrome, has been described in patients with acute neurological events [11]. At first, to meet the Initial Mayo Criteria for a positive TS diagnosis, an exclusion of serious head trauma and intracranial hemorrhage had to be established [12, 13]. But the 2008 revised Mayo Criteria no longer specified these acute neurological disorders as an exclusion criteria [4, 14]. Furthermore, Ghadri *et al*. [2] identified intracranial hemorrhage and other neurological instances as physical triggers in the development of TS. As a result, due to the similarities in pathophysiology, clinical and paraclinical features, we consider neurogenic cardiomyopathy as a type of TS [11].

Even though it was considered to be a self-limiting, benign disease, monitoring the TS patients in the acute and chronic phase revealed that short and long term outcome may be similar to acute coronary syndromes [3]. In the acute phase, electrical or hemodynamically instability may lead to important morbidity and mortality [15].

Two categories of Takotsubo patients have been suggested: primary and secondary [16]. The primary type is made up of mainly female patients who suffered an emotional trigger, developed mild systolic dysfunction and had a fast recovery. The secondary type does not have a female predominance, occurs after a physical trigger and has a more severe and/or prolonged myocardial dysfunction. But the main discrepancy between the two categories is the prognosis: primary TS patients have better outcome than those with secondary TS [16].

## 2. Pathophysiology

The pathophysiology of this syndrome is not entirely understood. Sympathetic stimulation is generally considered to play a major part, causing an adrenergic myocardial stunning. It is based on the patient’s secretion of adrenaline and noradrenaline and their effect upon the cardiovascular system [17]. One theory is that of an “aborted” myocardial infarction, with spontaneous lysis of the intracoronary thrombus. Another one is based on coronary spasm. But the constriction of a single coronary artery could not explain the ventricular motion abnormalities. That leaves the possibility of smaller vessels vasospasm and microcirculatory dysfunction. Coronary microvasculature may react to high levels of catecholamines and endothelin by transient vasoconstriction [2]. Currently Takotsubo syndrome is classified as an acute coronary syndrome due to microvascular dysfunction [18].

In most Takotsubo cases, a preceding physical or a psychological trigger can be identified. Ghadri *et al*. [2] illustrated a considerable list of triggers. Perhaps the recent pandemic revealed another possible TS trigger: coronavirus Disease (COVID)-19. Studies analyzing prepandemic and pandemic incidence of TS revealed a higher incidence in the COVID pandemic [19, 20]. The proposed mechanisms in COVID-19 patients are psychological stress associated with the disease, increased adrenergic response, cytokine storm and microvascular dysfunction. In the general population, social distancing and economic instability may be emotional factors leading to TS.

## 3. Diagnosis

The need to define and properly manage this disease led to the development of several diagnosic criteria, the most frequently used being the Mayo Clinical Criteria [14] and InterTAK Diagnostic Criteria [21]. The most common causes of hospital presentation are chest pain and dyspnea. Electrocardiographic changes are almost always present, with the most frequent one being ST segment elevation and/or T wave inversion [3]. Troponin levels in TS are usually elevated but maximum values do not generally reach those met in acute myocardial infarction [15]. A general opinion is that cardiac biomarkers (troponin and creatine kinase MB) are disproportionately low compared to the impaired wall motion [22]. The next step in the diagnostic algorithm is coronary angiography. Coronary disease is usually absent or can be present but discordant with the wall motion abnormalities. Subsequently, imaging methods bring to light the Takotsubo diagnosis.

## 4. Coronary and LV Angiography

Since the most frequent clinical presentation of a TS case is that of an acute coronary syndrome with ST segment elevation, the main investigation is coronary angiography in order to exclude a type 1 myocardial infarction [23, 24]. Coronary arteries need to be carefully assessed, in several angiographic views. Usually, patients with TS have non obstructive epicardial coronary arteries but coronary disease unrelated to the motion anomaly of the LV may be present. However, coronary artery disease is not an exclusion criteria [25]. The main reason is that the regional wall motion abnormalities (RWMA) extend beyond the vascularization of a single coronary artery [26]. Even if the patient has coronary artery disease, it is not an exclusion criteria if the depending myocardial territory is not concordant with the wall motion abnormalities [27, 28]. When coronary artery disease is found, orthogonal angiographic views of the artery and LV ventriculography help in observing the mismatch between the coronary stenosis and the wall motion abnormality. The “apical nipple sign” was described on ventriculography images: a small zone, right at the LV apex, with preserved contractility, found in approximately 30% of TS patients with apical pattern. This sign was not described in patients with acute anterior myocardial infarction and may help in differentiating the two conditions [29].

**Fig. 1.**
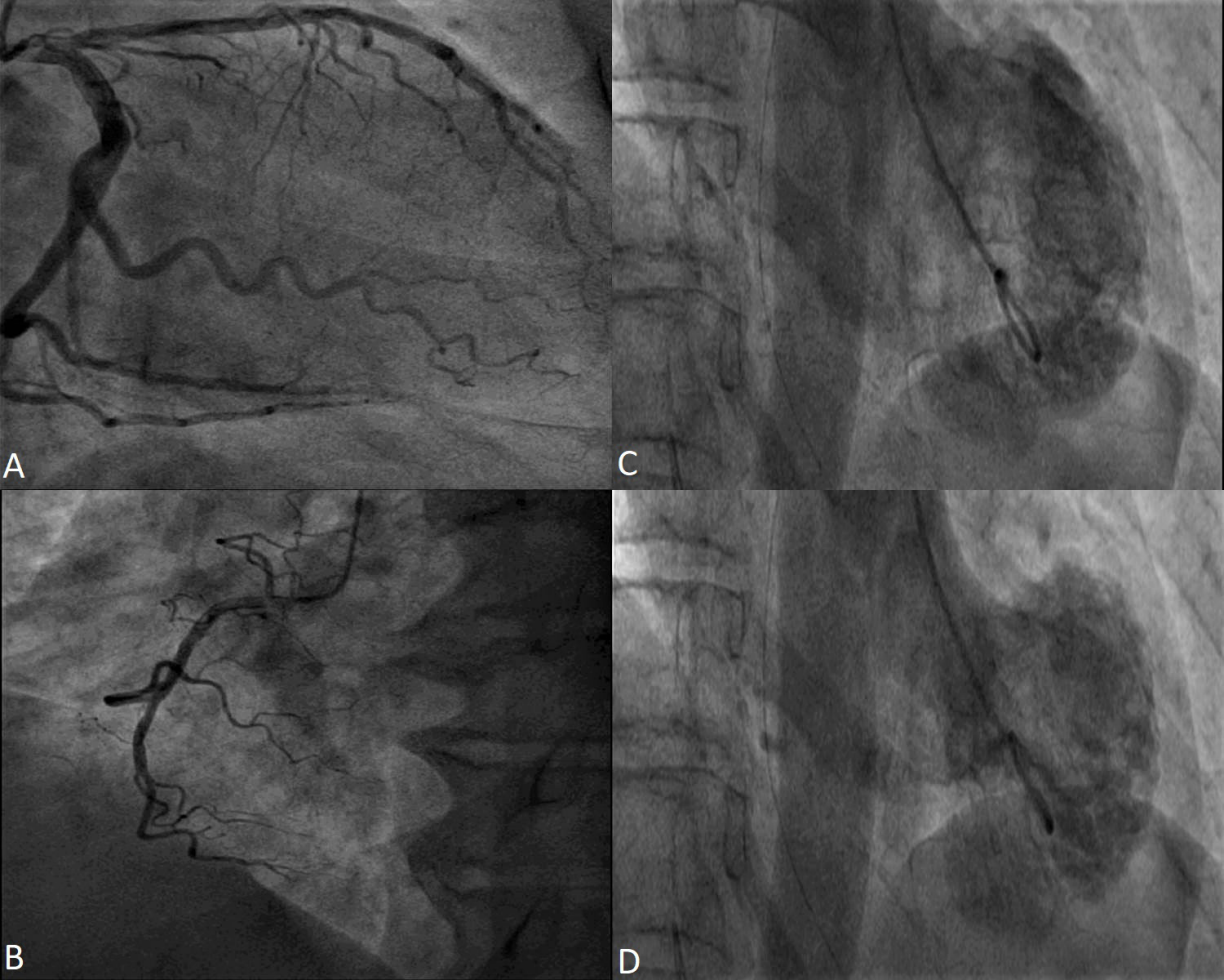
**Coronary angiography in a 53 year-old female patient with Takotsubo Syndrome.** Non-obstructive epicardial coronary arteries - images (A) and (B). Ventriculography showing apical akinesia and basal hyperkinesia - images (C) and (D).

Most of the time, TS diagnosis is associated with angiographically normal or non-obstructive coronary arteries [30] (Fig. 1). In patients diagnosed with TS and coronary artery disease, intracoronary imaging techniques, like intravascular ultrasound and optical coherence tomography, revealed that coronary plaques had no sign of thrombosis, erosion or dissection [31, 32, 33, 34].

In a lot of 11 consecutive patients admitted with acute coronary syndrome but later diagnosed with TS, the wall motion abnormalities were evaluated through ventriculography [35]. Unfortunately, the diagnostic workup did not include cardiac magnetic resonance (CMR) or patient follow-up after hospitalization. Even though ventriculography is a valuable tool in evaluating the circumferential pattern of ventricular motion abnormality when coronary angiography is performed, its usefulness is limited to the acute setting. Thus, in order to prove the reversible nature of the systolic dysfunction another imaging method, usually echocardiography, needs to be performed. In a retrospective study of 20 patients with TS, LV contractility was compared by assessing ventriculograms and strain derived echocardiography. The results suggest that echocardiography and strain analysis is the preferred modality in assessing the LV contractility [36].

Through catheterization, intracardiac pressures can be measured and certain complications can be detected, such as LV outflow tract obstruction (LVOTO). Elevated LV end diastolic pressure measured invasively during coronary angiography was described as a predictor of in-hospital complications [37].

Microvascular dysfunction is involved in the pathophysiology of TS. Angiography may be used in order to assess the coronary microvascular function. One of the methods is Thrombolysis in Myocardial Infarction (TIMI) frame count. Abnormal, diffusely reduced TIMI frame count values were described in TS patients compared to healthy subjects [38]. Abnormalities in microvascular circulation by altered TIMI flow grading were described in several studies [39, 40, 41]. Using an angiography-derived index of microcirculatory resistance (NH-IMRangio) during coronary angiography, proof of microvascular dysfunction was found in at least one of the coronary arteries in all 166 patients with TS [42].

## 5. Coronary Angiography by Computed Tomography

Assessment of the coronary arteries by coronary computed tomography angiography (CCTA) can be performed in patients with a high suspicion of TS and low likelihood of acute coronary syndrome [43, 44]. Furthermore, in patients with terminal disease or acute, important bleeding and a high suspicion of TS, physicians may opt for this non-invasive approach. Furthermore, another type of patients that could benefit from CCTA is those with acute neurological events, especially in subarachnoid hemorrhage. Young patients with no cardiovascular risk factors, low ischemic risk and a high suspicion of TS, CCTA might be better suited for a non-invasive management. Assessing the coronaries by CCTA may also have a role in recurrent TS [3]. In case of acute hemodynamic instability of uncertain etiology, assessment by computed tomography can analyze other sections in order to exclude pulmonary embolism and acute aortic syndromes [44].

## 6. Standard Echocardiography

Transthoracic echocardiography (TTE) is an easily accessible and reproductive method available from the Emergency Department in order to assess patients with suspected acute coronary syndrome or acute heart failure. It has an essential role in the management of TS cases.

**Fig. 2.**
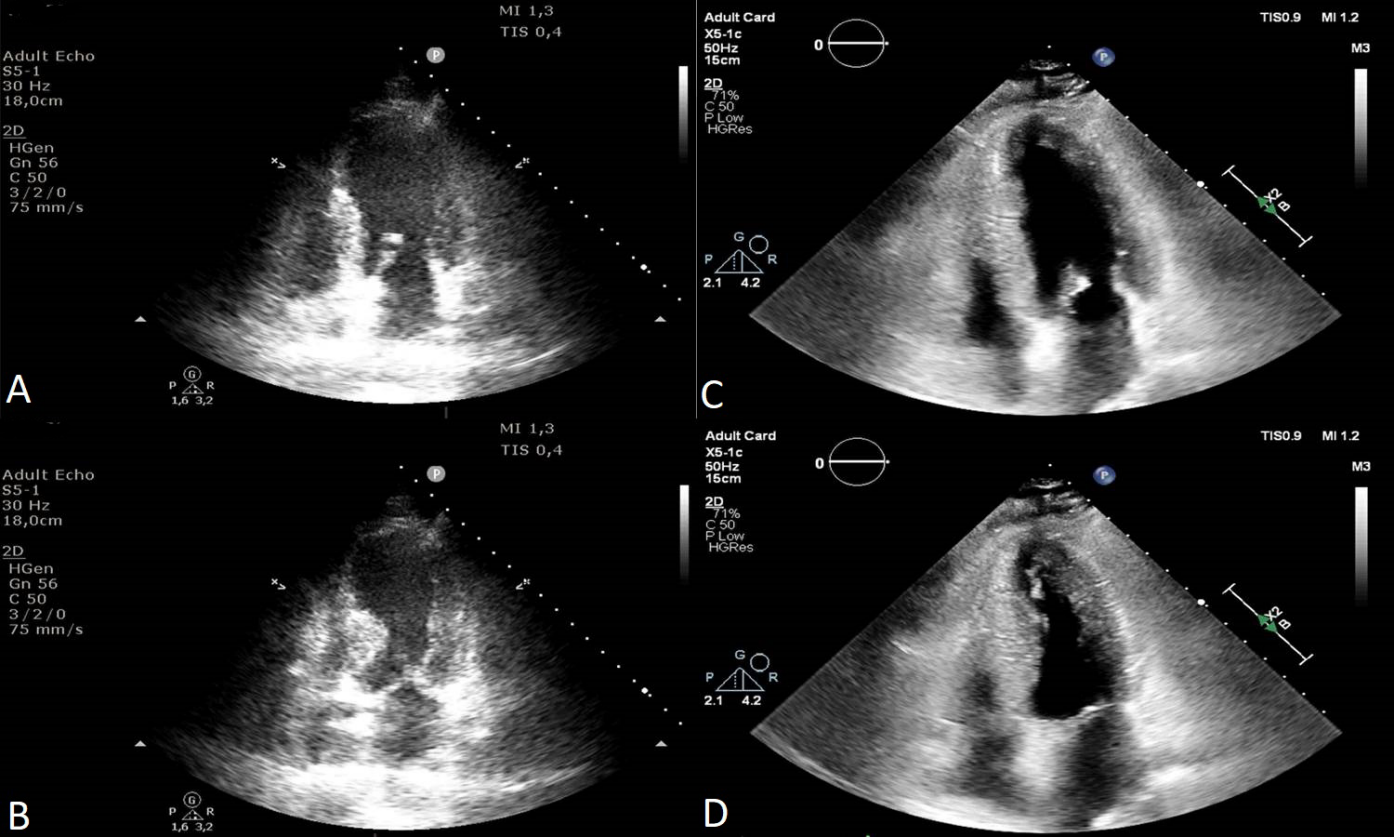
**Transthoracic echocardiography images apical four-chambers views of a 73 year-old female patient with Takotsubo Syndrome.** Apical akinesia in the acute phase (A and B) and normalized wall motion and ejection fraction at 1 month follow-up (C and D).

It is mainly used in documenting LV systolic and diastolic function [45]. The RWMA is usually of a circumferential pattern and extend beyond the distribution of one coronary artery. The most frequent phenotype is the apical pattern — akinesia of the apical segments of the LV with basal compensatory hyperkinesis (Fig. 2). Atypical patterns are those affecting medioventricular, basal or focal myocardial territory [46, 47, 48, 49]. Case reports have described isolated right ventricular (RV) Takotsubo [50, 51].

Another important role of TTE is in patients with acute heart failure, exhibiting as acute pulmonary edema or cardiogenic shock. Assessing the biventricular function can point towards the cause of cardiac decompensation.

TTE aides in identifying TS complications (Table 1). The hyperkinesia of basal segments, sometimes associated with a septal bulging can lead to LVOTO, with or without systolic anterior motion (SAM) of the anterior mitral leaflet [52]. Mitral regurgitation can be caused by leaflet tethering due to geometrical changes in the LV (according to TS pattern) and the displacement of the papillary muscles or by SAM [53, 54]. Although a rare instance, LV free wall or interventricular septal rupture may occur [55, 56, 57, 58, 59]. Apical akinesia may promote intraventricular thrombosis and furthermore thromboembolic events may occur [56, 60]. The development of ventricular thrombosis is more frequent in older patients and in those with late presentation to the hospital [61]. Contrast echography may help in patients with poor acoustic window in identifying motion abnormalities and intraventricular thrombosis.

**Table 1.** **Takotsubo Cardiomyopathy complications diagnosed by echocardiography**.

Severe systolic dysfunction
Left ventricular tract obstruction
Moderate to severe mitral regurgitation (with or without systolic anterior motion)
Apical thrombosis
Ventricular septal defect
Free wall rupture
Right ventricular involvement
Pericardial Effusion

Cardiogenic shock in TS can be caused by severe systolic dysfunction, LVOTO, severe mitral regurgitation, and RV involvement [52, 62, 63]. Diagnosing TS complications is of utmost importance in order to properly treat an individual patient. TTE is particularly useful in diagnosing TS in acute clinical scenarios which are not suggestive of a cardiac event [64], such as patients with neurological disease who also present dynamic electrocardiogram (ECG) changes and elevated troponin levels [65, 66, 67, 68]. TTE has an important role in the perioperative and periprocedural care or in patients who become hemodynamically unstable in the Intensive Care Unit [48, 69, 70, 71, 72, 73, 74]. Bedside echocardiography in the Intensive Care Unit plays an important part in differentiating among different types of hemodynamic instability [9, 75].

But the role of ultrasound imaging does not end here. Through its definition, TS is a reversible systolic dysfunction, thus extending the role of echocardiography from diagnosis to follow-up [76, 77] [Fig. 2]. It is necessary to document the recovery of the ventricular systolic function –and the progression or regression of complications [54, 78]. As diagnostic Takotsubo Criteria mention, the wall motion anomalies are transient [2, 4, 5]. As a result, echocardiographic follow-up is mandatory, especially if the patients cannot be evaluated by CMR. As demonstrated in this thorough study, the LV systolic function improves in the first 2 weeks and continues to recover over the next 4 weeks [79]. Documenting the left ventricular ejection fraction (LVEF) also has prognostic implications. Reduced ejection fraction (≤35%) is a poor prognostic marker for both short and long term prognosis [80].

**Fig. 3.**
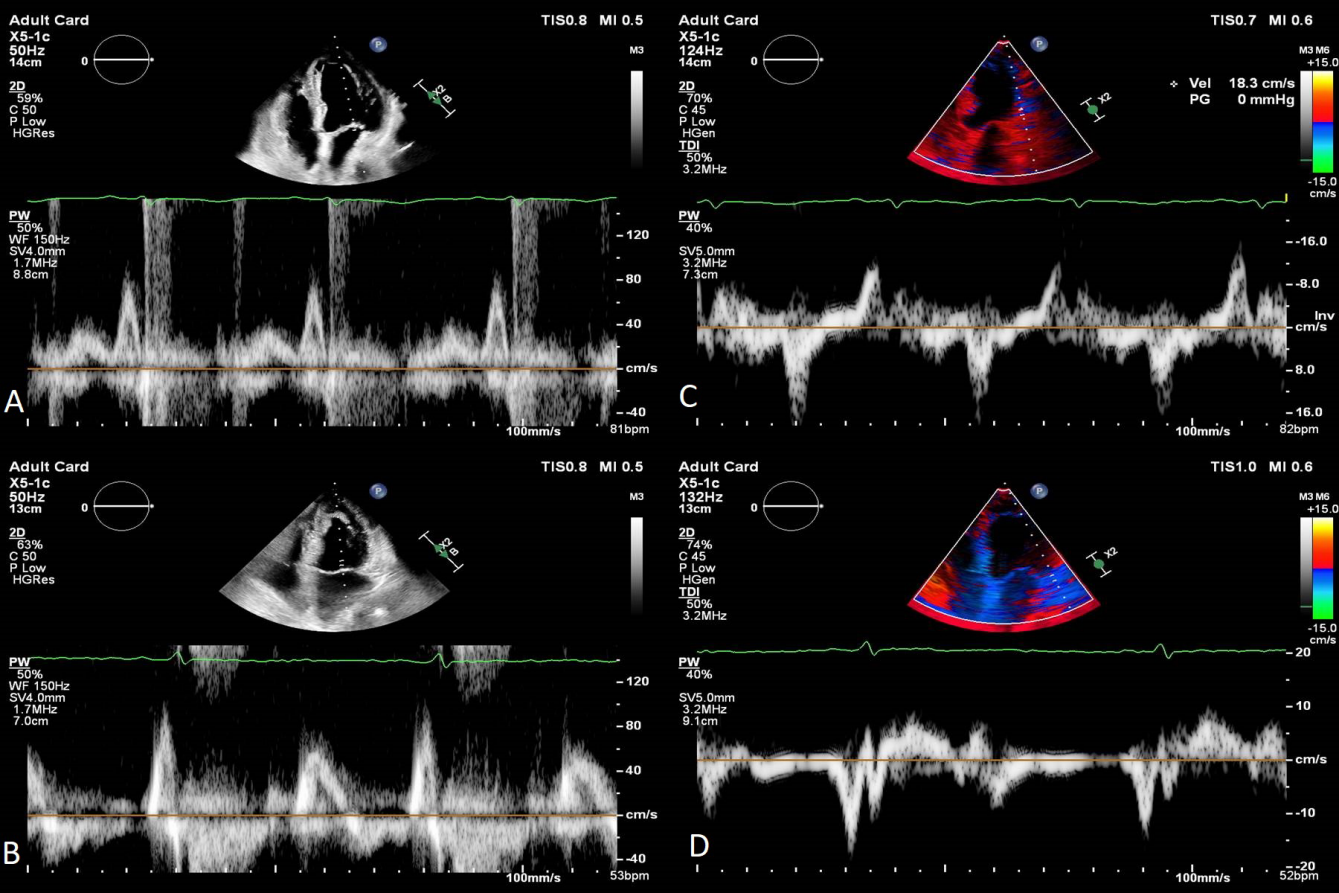
**Transthoracic echocardiography images apical four-chambers views of a 73 year-old female patient with Takotsubo Syndrome showing the evolution of diastolic function. **E wave velocity progressed from 40 cm/s in the acute phase to 70 cm/s at one month follow-up, the E/A ratio from 0.5 to 0.8 (A and B). Lateral e’ velocity increased from 4 cm/s to 8 cm/s and E/e’ ratio from 10 to 8.7 (C and D). 2D, two dimensional; PW, pulsed wave doppler; TDI, tissue doppler imaging.

Alongside the LV systolic function, LV diastolic function needs to be evaluated in the acute phase and then at follow-up. Kumar *et al*. [81] revealed that LVEF improved alongside diastolic function: E value, E/A ratio and e’ values improved over time (Fig. 3). Both lower LVEF and higher E/e’ ratio were associated with in-hospital complications in TS patients [37, 54].

Tei Index, a combined indicator of systolic and diastolic function, has limited value in Takotsubo cases. Mirna *et al*. [82] showed that Takotsubo patients had higher Tei Index as compared to patients with an acute coronary syndrome and to controls.

Surprisingly, three dimensional (3D) echocardiography has not yet been considerably used to investigate patients with TS. It might assist in a more thorough assessment of the left and right ventricle and their function [83].

## 7. Speckle Tracking Echocardiography

**Fig. 4.**
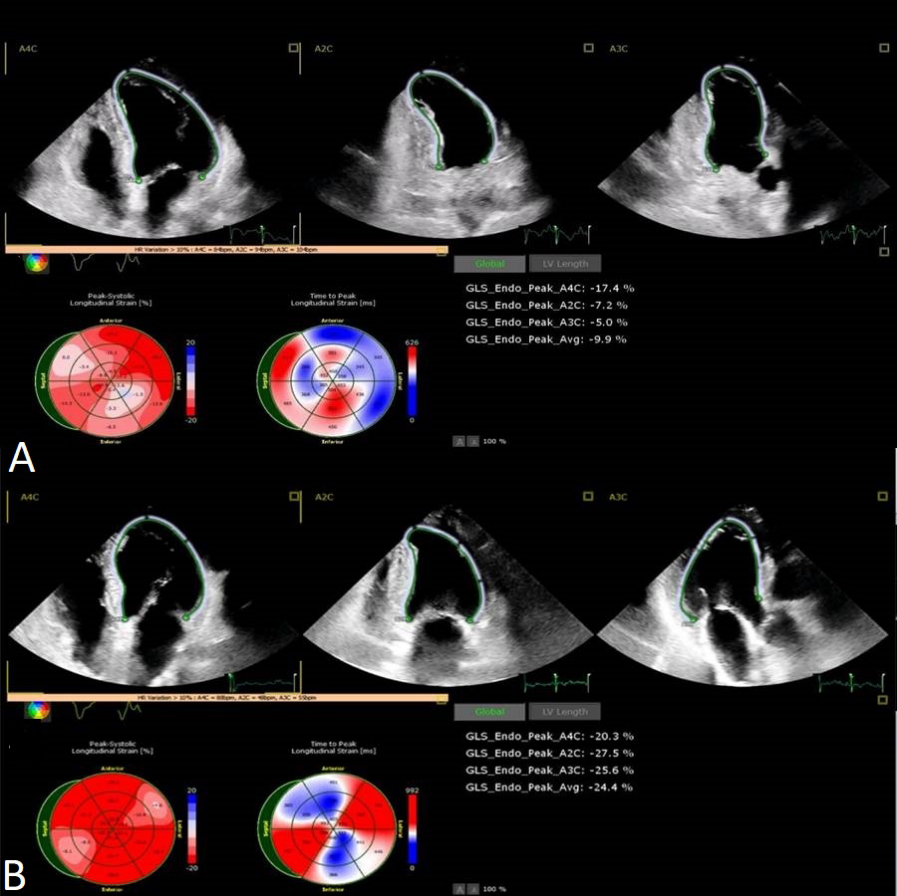
**Speckle tracking echocardiography showing the improvement in global longitudinal strain and time to peak longitudinal strain from the acute phase (A) to 1 month follow-up (B).** GLS, global longitudinal strain.

Strain TTE is a rigorous method of evaluating regional and global left and right myocardial function. It is angle independent and it requires a proper acoustic window. Strain images unfolded important information about patients with TS. By speckle tracking analysis, the systolic function of the left ventricle had a more delayed recovery compared to the calculated LV ejection fraction [28, 84, 85, 86]. Even though it is generally accepted that in the apical and midventricular variant the base of the LV is hyperkinetic, strain analysis revealed contractility impairment even in the “hyperkinetic” areas [36, 87]. LV strain assessment has prognostic value in the acute phase of TS patients [88]. In TS, the motion abnormalities are not subtle, especially in the apical variant. As a result, speckle tracking echography has a more pronounced role in the follow-up part rather than in the acute, diagnostic part. Kobayashi *et al*. [85] performed follow-up 3D strain echography at 4 weeks and 6 months after the diagnosis. They identified that regional abnormalities by peak systolic shortening and peak systolic thickening persisted at 4 weeks despite normalized LVEF (Fig. 4). The persistence of myocardial dysfunction assessed by LV strain was described at 4 weeks follow-up in Takotsubo patients [89].

Speckle tracking echography showed impaired of LV contraction and relaxation by analyzing peak systolic strain rate and early diastolic strain rate, which both improved over time [78].

In a case control study, patients with a history of TS (over 12 months prior to enrollment) were evaluated by echocardiography and CMR and compared to matched control subjects [90]. There were no differences between LVEF calculated by echocardiography and CMR, but the patients with prior Takotsubo presented impaired left ventricular longitudinal and circumferential strain, which highlights the idea that TS is not a “benign” syndrome.

Despite all the advances in ultrasound imaging, echocardiography cannot differentiate between TS and acute coronary syndrome in the acute phase, thus placing coronary angiography as a major investigation in Takotsubo diagnosis [91].

## 8. Dobutamine Stress Echocardiography and Takotsubo Syndrome

Dobutamine and TS have a convoluted rapport. A few cases of TS during or immediately after Dobutamine stress Echography have been reported [92, 93, 94, 95, 96, 97]. Coronary angiography excluded coronary artery disease in all of these patients and complete recovery of the LV systolic function was noted on follow-up echocardiography. These cases of TS in the context of Dobutamine stress test may support the important part that catecholamines play in the pathophysiology of this disease.

In a Dobutamine Stress Study in which 22 patients with a history of Takotsubo (6 months after the diagnosis) were compared to 22 control subjects, no patient developed wall motion abnormalities suggesting that the susceptibility to adrenergic stimulation did not persist after the initial diagnosis [34]. Stress echography was also used in the acute phase of TS in order to unmask LVOTO. The study emphasized the need for beta blockers in patients with LVOTO or who developed LVOTO but safety of the investigation is not yet defined.

## 9. Contrast Echocardiography

The main utility of contrast TTE is the evaluation of the distribution of left and right ventricle wall motion abnormalities in patients with TS and poor acoustic window. Furthermore, it is useful in detecting intraventricular thrombosis. Contrast echocardiography was used in order to reveal myocardial perfusion defects in the affected areas suggesting transient microvascular dysfunction [98, 99].

## 10. Transthoracic Coronary Artery Doppler

In patients with a good acoustic window, assessment of left anterior coronary Doppler by TTE can help differentiate between acute anterior myocardial infarction and TS — apical variant [100]. Pulsed wave Doppler during dypiridamole echography tests showed a decrease in the coronary flow reserve in the early days of TS and recovers alongside the systolic function [101].

## 11. Cardiac Magnetic Resonance

CMR has a developing role in the diagnosis of TS [102]. It is a valuable tool in differentiating between TS and other causes in patients presenting with chest pain, elevated troponin levels and coronary arteries with no significant stenoses. CMR provides a dynamic and structural assessment of the myocardium and also detects myocardial inflammation and scarring [103].

In the acute phase, the cine sequences of CMR allow the visualization of RWMA (apical, midventricular), the circumferential pattern, the RV involvement [47]. It can also show complications: pericardial effusion, systolic anterior movement mitral with mitral regurgitation, ventricular thrombosis. Maybe most importantly, CMR provides tissue characterization — it can detect myocardial edema, necrosis, fibrosis — essential criteria for the differential diagnosis with myocardial infarction and myocarditis [103, 104].

In the Stockholm Myocardial Infarction with Normal Coronaries Study I and II, patients diagnosed with myocardial infarction with non-obstructive coronary arteries (MINOCA) underwent CMR evaluation. Among them, approximately a third were diagnosed with TS, differentiating them from patients with myocardial infarction, myocarditis or other cardiomyopathies (dilated, hypertrophic cardiomyopathy) [105, 106]. CMR is superior in detecting RV involvement than echocardiography and in evaluating the RV function.

**Fig. 5.**
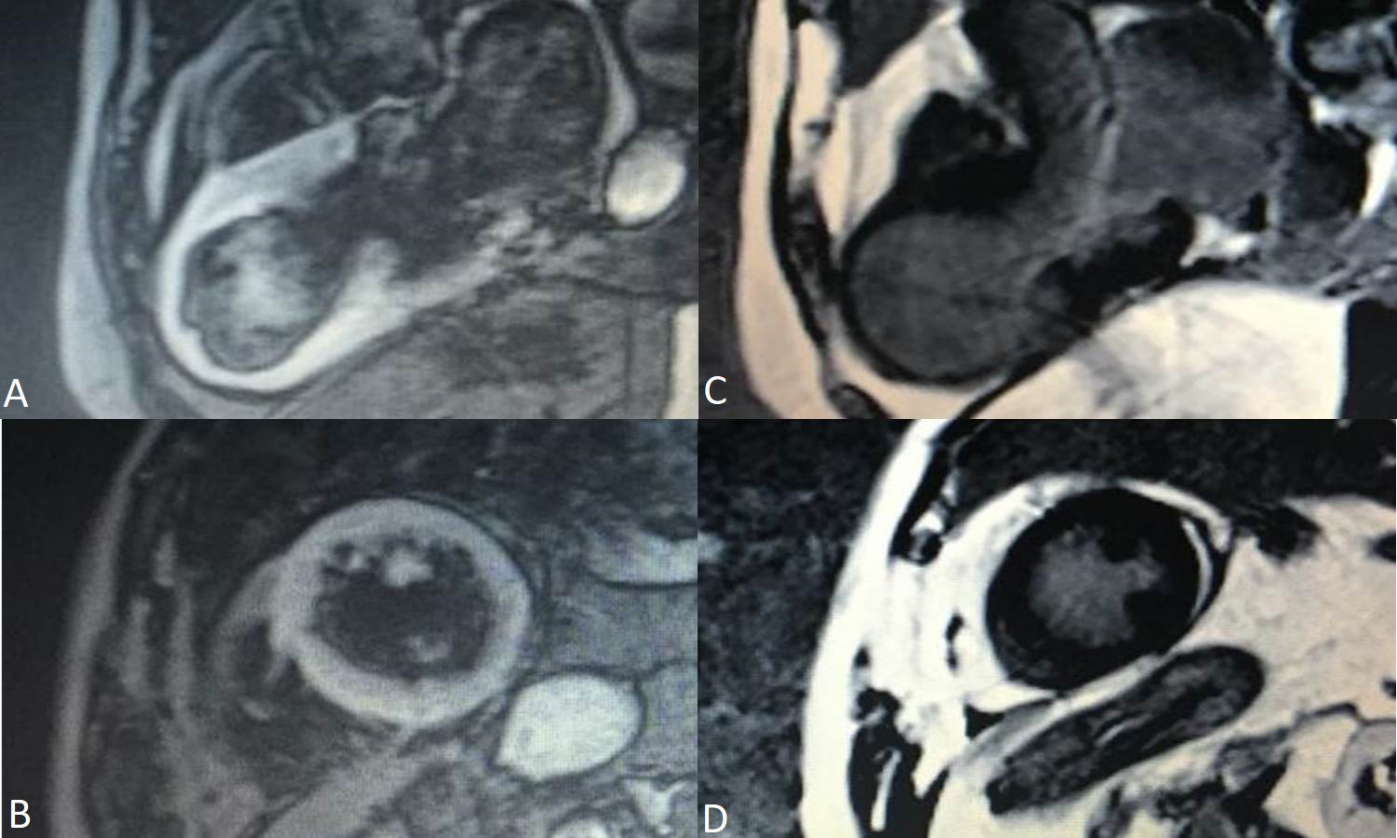
**Cardiac Magnetic Resonance Imaging of a 78 years old female patient in the acute phase of Takotsubo Syndrome.** Myocardial edema can be observed in the apical regions of the left ventricle in T2 weighted images (A and B) and the lack of late gadolinium enhancement in C and D.

CMR using T2 weighted images is a noninvasive alternative in discovering myocardial edema. The areas of myocardial edema correlated with RWMA areas [107, 108] (Fig. 5). Studies who performed endomyocardial biopsies in Takotsubo patients proved there is inflammation in the motion abnormalities areas [109].

Generally, there is no fibrosis detected on late gadolinium enhancement (LGE) CMR. In the chronic phase, CMR follow up reveals normal ventricular function, normal regional wall motion, no edema, no necrosis and no fibrosis [110]. Even though in most Takotsubo cases there was no LGE present, LGE positive cardiac magnetic Resonance imaging (MRI) in TS were reported in a few cases [111, 112, 113]. A two-step recovery in the LV function in TS was described. The first step was the recovery of the LV systolic function and the latter one was the improvement of the diastolic one, assessed by LV peak filling rates and left atrial filling volumes [114]. CMR based strain analysis of the LV in the acute phase revealed that CMR does have prognostic value in TS patients. However, prognosis was mainly influenced by the patients’ comorbidities [115].

Studies that enrolled patients with suspected TS patients performed CMR in order to establish the diagnosis. Thirty-seven patients with RWMA, non-obstructive coronary arteries, ECG abnormalities and elevated troponin underwent CMR evaluation. Four patients were diagnosed with myocarditis, 7 patients with myocardial infarction and the rest [26] were diagnosed with TS [110]. During the COVID-19 pandemic, there were a few cases of vaccine associated myocarditis reported [116]. In one particular case, CMR aided the medical team in discovering a case of vaccine associated TS [117].

Even though echocardiography is a an extremely valuable tool in the diagnosis and management of TS, only CMR and endomyocardial biopsy can truly distinguish between myocarditis and TS [118].

In the rare instances of acute myocardial infarction and TS simultaneity, CMR is the only exploration able to describe both areas of myocardial edema suggestive of TS and areas of subendocardial or transmural ischemic necrosis [119, 120].

Regarding female patients in the peripartum stage, acute systolic dysfunction requires a difficult differential diagnosis, including peripartum cardiomyopathy, acute myocarditis, TS, acute myocardial infarction and genetic cardiomyopathy. In order to differentiate among these, CMR needs to be performed [121].

**Fig. 6.**
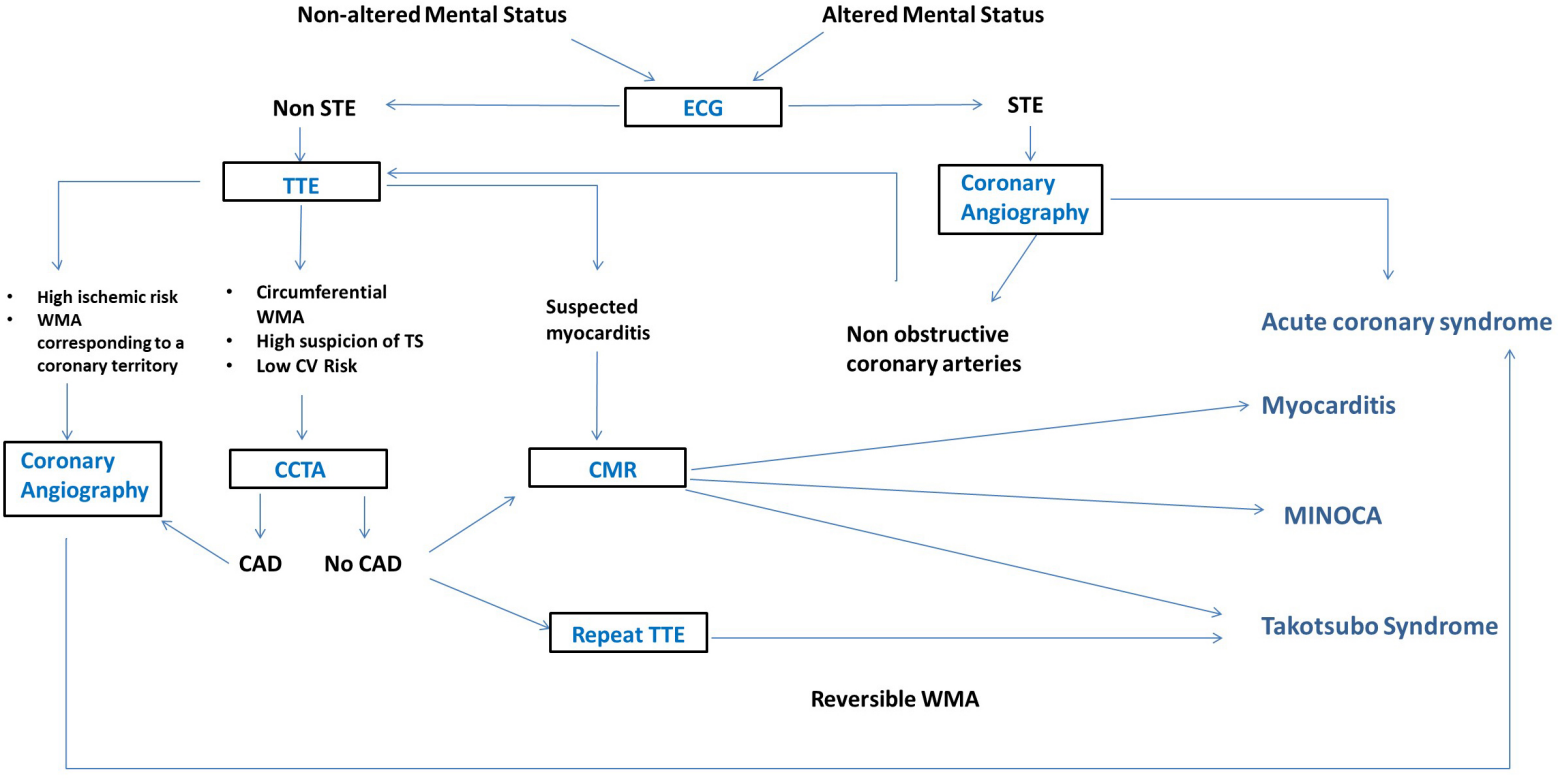
**Imagistic Diagnostic Flowchart of Takotsubo Syndrome (Modified after Ghadri *et al*. 2018 [3]).** CAD, coronary artery disease; CMR, cardiac magnetic resonance; ECG, electrocardiogram; MINOCA, Myocardial infarction with non-obstructive coronary arteries; STE, ST segment elevation; TTE, transthoracic echocardiography; WMA, wall motion abnormalities; CCTA, coronary computed tomography angiography; TS, Takotsubo syndrome; CV, cardiovascular.

When patients present with apical RWMA and non-obstructive coronary arteries, the clinical likelihood of TS is high. But when a patient presents with an atypical pattern, the TS diagnosis is so much more difficult. In such cases, CMR is of unquestionable benefit [122] (Table 2). The positive and differential diagnosis in TS is a complex one, starting from the clinical arguments of TS likelihood and ischemic risk, and oftentimes extending beyond echocardiography and coronary artery imaging to magnetic resonance myocardial description (Fig. 6).

**Table 2.** **The main imaging methods in Takotsubo Cardiomyopathy — positive and negative points**.

Takotsubo Cardiomyopathy					
Echocardiography		Cardiac Magnetic Resonance		Coronary Angiography	
+	–	+	–	+	–
∙ Easily accessible	∙ Acoustic window dependent	∙ LV/RV function	∙ High cost	∙ LV/RV function	∙ Invasive
∙ LV/RV function		∙ Tissue characterization		∙ Differential diagnosis with coronary artery disease	∙ Radiation exposure
∙ Complications		∙ Complications			∙ No follow-up
∙ Follow-up		∙ Follow-up			
		∙ Differential diagnosis			

LV, left ventricle; RV, right ventricle.

## 12. Nuclear Imaging

Nuclear imaging techniques in TS patients have a limited role in clinical practice. Their importance resides in deepening the understanding of TS’s pathophysiology. Nuclear techniquesare used in investigating the myocardial perfusion and myocardial metabolism (using metabolites such as fatty acids and glucose). Studies using positron emitted tomography with fluorodeoxyglucose and single-photon emission computerized tomography with fatty acids revealed both metabolic and perfusion abnormalities but a more profound defect was found in the metabolic activity compared to the perfusion defect [39, 123, 124]. This imbalance found in the wall motion abnormalities was named “inverse perfusion-metabolism mismatch”. Impaired perfusion suggests microvascular dysfunction.

Myocardial scintigraphy using ^123^I Metaiodobenzylguanidine (a molecule which resembles noradrenaline structurally) revealed a cardiac adrenergic dysfunction in the acute phase in the motion abnormalities areas, which improved over time [39, 125]. These methods of sympathetic nervous imaging support the idea of adrenergic mediated myocardial stunning.

## 13. Conclusions

Takotsubo cardiomyopathy is a condition with a wide variety of patterns and complications. Even though the reversibility of TS suggests a “benign” course, over the years many complications and poor long term prognosis were described. The complex management starts with the diagnosis and ends with a not so favorable prognosis. Echocardiography is the paramount investigation used in the diagnosis and follow up of the syndrome. Coronary angiography is of utmost importance when suspecting an acute coronary syndrome that needs urgent revascularization. CMR is most useful in the differential diagnosis with other types of acute systolic dysfunction, ischemic or non-ischemic. Strain assessment by echocardiography and CMR may provide valuable prognostic information in the near future.
